# Supplementary material for: Magnetic Resonance Imaging (MRI) of Spanish Sheep Cheese: A Study on the Relationships between Ripening Times, Geographical Origins, Textural Parameters, and MRI Parameters
Source: Foods. 2024 Oct 10;13(20):3225. doi: 10.3390/foods13203225 (PMC11507292; doi:10.3390/foods13203225)
Supplement: Supplementary file 1 [file foods-13-03225-s001.zip › foods-3127104-supplementary.pdf]

Table S1. Manufacture characteristics of the studied varieties of Spanish sheep cheese.

		I-CLM	T-CLM	I-CL	T-CL
**Production location**		Castilla-La-Mancha (Central Spain)	Castilla-La-Mancha (Central Spain)	Castilla y León (north-western Spain)	Castilla y León (north-western Spain)
**Manufacture**		In large enterprises, according to PDO Manchego cheese	In local small enterprises according to traditional manufacture of ewe´s milk cheese	In large enterprises, according to Castellano cheese quality mark	In local small enterprises according to traditional manufacture of ewe´s milk cheese
**Commercial designation**		Manchego cheese PDO	Traditional Manchego sheep cheese	Castellano sheep cheese	Traditional Castellano sheep cheese
**Sheep breed**		Manchega	Manchega, Merina and Talaverana	Castellana and Churra	Castellana and Churra
**Milk Pre-treatment**		Pasteurised	Raw	Raw	Raw
**Starter Culture**		Homo-fermentative mesophilic LAB	Homo-fermentative mesophilic LAB	Homo-fermentative mesophilic LAB	Homo-fermentative mesophilic LAB
**Rennet**		Recombinant Chymosin	Calf commercial dried rennet	Recombinant Chymosin	Lamb commercial natural rennet
**Coagulation**	**T (°C)**	30-32	30-32	32	28-32
	**Time (min)**	45	40-45	35-40	30-45
**Cutting Curd**		Continuous automated process	Semi-continuous process	Continuous automated process	Semi-continuous process
	**Grain size (mm)**	5-10	5-10	5-8	5-10
**Mild stirring**		Continuous automated process	Discontinuous process	Continuous automated process	Discontinuous process
**Pre-drainage of whey**		Continuous automated process (mild stirred and pumped whey)	Manual (whey drainage by soft pressing)	Semi-continuous processes (mild stirred and pumped whey)	Manual (whey drainage by soft pressing)
**Scalding**	**T (°C)**	36-40	34-38	35-38	35-40
	**Time (min)**	30-35	30-40	40	30-40
**Pre-pressing**		Continuous automated process (previous pressing depending on mold size)	Manual (in curdling vats)	Continuous automated process (in curdling vats)	Manual (in curdling vats)
	**T (°C)**	30-32	30-32	30-32	30-32
	**Time (min)**	35	30	30	30
**Moulding**		Automatic	Manual	Manual	Manual
**Pressing**		Through pneumatic press	Through pneumatic press	Through pneumatic press	Through pneumatic press
	**Time (h)**	4	10-12	10-12	10
**Unmoulding**		Automated	Manual	Automated	Manual
**Salting**		By immersion in sodium chloride (20 g /100 mL)	By immersion in sodium chloride (23 g /100 ml)	By immersion in sodium chloride (20-22 g/100 mL)	By immersion in sodium chloride (20-21 g/100 mL)
	**T (°C)**	10	10-15	10	10-15
	**Time (min)**	20-24	20-24	24-30	28-32
**Ripening**		In chambers with automated control of temperature and RH	In chambers with automated control of temperature and RH	In chambers with automated control of temperature and RH	In chambers under natural conditions
	**T (°C)**	8-10	10	10	10-12
	**RH (%)**	80	75-80	75-80	75-80
	**Time (days)**	60 days for pieces bigger than 1.5 kg<br>Semi-cured: 60-90 days<br>Cured: 180 days			

I-CLM = pieces produced in an industry in Castilla-La-Mancha region; T-CLM = pieces manufactured according to an artisanal/traditional procedure also in Castilla-La-Mancha; I-CL = pieces produced in a large industry in Castilla y León; T-CL = manufactured according to an artisanal/traditional procedure also in Castilla y León. PDO = protected designation of origin. LAB = lactic acid bacteria. T (°C) = temperature; RH = relative humidity (%).

Table S2. Values of the physicochemical features (pH, a_w, DM, WC, Ash, Fat, and Protein contents) of the four considered Spanish sheep cheese varieties at different ripening times.

	RT* (days)	I-CLM#			T-CLM			I-CL			T-CL		
	2	5.69±0.04	a	α	5.58±0.03	a	β	5.26±0.03	ab	γ	5.18±0.06	a	γ
	9	5.66±0.06	a	α	5.19±0.06	b	γ	5.33±0.04	a	β	5.06±0.05	b	γ
**pH**	30	5.44±0.02	b	α	5.14±0.05	b	βγ	5.22±0.03	b	β	5.07±0.02	b	γ
	90	5.48±0.01	b	α	5.20±0.02	b	β	5.23±0.01	b	β	5.11±0.02	ab	γ
	180	5.49±0.02	b	α	5.13±0.01	b	γ	5.30±0.01	ab	β	5.11±0.04	ab	γ
	2	0.952±0.001	a	γ	0.961±0.001	a	α	0.957±0.002	a	β	0.964±0.003	a	α
	9	0.951±0.002	a	α	0.954±0.002	ab	α	0.952±0.001	a	α	0.945±0.008	b	β
a_w	30	0.942±0.007	b	α	0.946±0.003	bc	α	0.946±0.003	b	α	0.929±0.001	c	β
	90	0.936±0.002	bc	α	0.942±0.007	c	α	0.911±0.009	c	γ	0.926±0.002	c	β
	180	0.933±0.003	c	α	0.904±0.002	d	β	0.897±0.009	d	γ	0.891±0.002	d	γ
	2	50.85±0.177	d	β	51.92±2.638	c	α	50.82±0.493	c	β	52.91±0.258	d	α
**DM** (%,	9	51.59±0.718	d	β	53.61±1.446	c	α	52.49±1.047	c	β	54.73±0.746	c	α
w/w of	30	58.06±0.389	c	β	57.07±0.854	b	γ	60.33±1.726	b	α	58.38±0.413	b	β
cheese)	90	63.00±0.994	b	αβ	60.88±1.038	a	β	65.97±0.506	a	α	61.56±0.783	a	β
	180	67.70±0.835	a	α	62.51±1.810	a	β	66.19±0.485	a	α	63.09±0.732	a	β
	2	49.15±0.176	a	α	48.07±2.638	a	β	49.17±0.493	a	α	47.08±0.257	a	β
	9	48.41±0.717	a	α	46.38±3.866	a	β	47.50±1.046	a	α	45.27±0.746	b	β
**WC** (%)	30	41.94±0.389	b	β	42.99±0.854	b	α	39.66±1.726	b	γ	41.61±0.413	c	β
	90	36.99±0.994	c	αβ	39.11±1.038	c	α	34.02±0.505	c	β	38.44±0.783	d	α
	180	33.29±0.835	d	β	37.48±1.810	c	α	33.81±0.484	c	β	36.90±0.732	d	α
	2	6.01±0.09	c	β	6.33±0.24	b	β	7.56±0.34	b	α	6.93±0.47	b	α
**Ash** (%,	9	6.11±0.13	c	β	6.67±0.19	b	β	7.71±0.48	b	α	7.06±0.27	b	α
w/w of	30	6.17±0.06	bc	β	6.57±0.51	b	αβ	7.39±0.72	b	α	6.99±0.27	b	α
DM)	90	6.49±0.29	b	β	6.69±0.18	b	β	7.65±0.12	b	α	6.95±0.55	b	β
	180	8.38±0.27	a	α	7.33±0.46	a	β	8.40±0.23	a	α	8.01±0.22	a	αβ
	2	50.3±2.15	a	β	54.4±2.89	a	αβ	55.3±0.78	a	α	55.2±1.32	a	α
**Fat** (%,	9	51.9±1.60	a	α	53.9±1.27	a	α	54.6±1.48	a	α	54.4±1.09	a	α
w/w of	30	51.9±0.72	a	β	53.9±1.31	a	αβ	54.1±1.17	a	α	54.1±1.31	a	α
DM)	90	50.7±0.43	a	β	52.3±3.70	a	αβ	55.1±1.24	a	α	54.6±3.32	a	α
	180	52.1±0.89	a	β	52.8±1.61	a	αβ	54.3±1.06	a	α	54.1±1.21	a	α
	2	35.8±1.94	a	α	33.7±0.92	b	α	33.2±0.56	a	αβ	32.0±1.29	b	αβ
**Protein**	9	36.7±1.89	a	α	33.0±1.04	b	β	34.6±1.12	a	αβ	33.1±0.83	ab	β
(%, w/w	30	36.9±0.99	a	α	33.6±0.54	b	β	34.2±1.37	a	αβ	33.9±0.74	a	β
of DM)	90	36.2±3.26	a	αβ	37.3±1.53	a	α	33.9±1.04	a	β	34.4±1.01	a	β
	180	37.2±0.89	a	α	37.8±1.44	a	α	34.5±1.48	a	β	35.0±0.86	a	β

a, b, c, d...: Different letter within a column for the same physicochemical characteristic indicates statistically significant differences ($p < 0.05$) related to ripening time.

α, β, γ...: Different letter within a row indicates statistically significant differences ($p < 0.05$) related to the cheese variety.

*RT = ripening time (days); DM = dry matter (%); WC = water content (%, 100-DM).

#Cheese variety (I-CLM, T-CLM, I-CL, and T-CL) according to Table S1.

Table S3. Regression parameters of the linear models between MRI parameters and physicochemical and texture parameters.

			I-CLM[a]			T-CLM			I-CL			T-CL		
			Coefficient	SD[b]	*p*-value	Coefficient	SD	*p*-value	Coefficient	SD	*p*-value	Coefficient	SD	*p*-value
RT (days)[c]	T_1	Intercept	15.03	0.4060	0.0001	19.90	0.8927	0.0001	18.05	1.2064	0.0001	15.42	0.5245	0.0001
		Slope	-0.034	0.0010	0.0001	-0.042	0.0020	0.0001	-0.029	0.0020	0.0001	-0.021	0.0009	0.0001
	T_2	Intercept	12.59	0.7771	0.0001	10.62	0.4847	0.0001	-5.762	0.1870	0.0001	-0.320	0.2463	0.1995
		Slope	-0.235	0.0161	0.0001	-0.275	0.0132	0.0001	0.133	0.0036	0.0001	0.090	0.0063	0.0001
a_w	T_1	Intercept	0.880	0.0078	0.0001	0.733	0.0238	0.0001	0.705	0.0547	0.0001	0.752	0.0175	0.0001
		Slope	0.000	0.0000	0.0001	0.000	0.0001	0.0001	0.000	0.0001	0.0003	0.000	0.0000	0.0001
	T_2	Intercept	0.898	0.0088	0.0001	0.828	0.0105	0.0001	1.034	0.0170	0.0001	0.984	0.0035	0.0001
		Slope	0.001	0.0002	0.0001	0.003	0.0003	0.0001	-0.002	0.0003	0.0001	-0.001	0.0001	0.0001
WC (%)[d]	T_1	Intercept	-8.146	4.8019	0.1009	-5.426	5.2999	0.3147	-32.07	12.3685	0.0150	11.36	2.0336	0.0001
		Slope	0.125	0.0119	0.0001	0.106	0.0116	0.0001	0.123	0.0209	0.0001	0.052	0.0034	0.0001
	T_2	Intercept	7.164	6.2550	0.2618	20.54	3.4713	0.0001	74.17	2.5022	0.0001	49.61	0.9112	0.0001
		Slope	0.726	0.1294	0.0001	0.617	0.0949	0.0001	-0.648	0.0478	0.0001	-0.215	0.0232	0.0001
Hardness (N)	T_1	Intercept	81.30	7.2964	0.0001	84.61	10.285	0.0001	77.06	8.2174	0.0001	80.47	4.7258	0.0001
		Slope	-0.128	0.0182	0.0001	-0.130	0.0225	0.0001	-0.095	0.0138	0.0001	-0.096	0.0079	0.0001
	T_2	Intercept	72.60	6.9519	0.0001	63.90	4.2113	0.0001	0.833	3.2176	0.7967	12.14	2.1592	0.0001
		Slope	-0.882	0.1440	0.0001	-1.060	0.1145	0.0001	0.381	0.0616	0.0001	0.323	0.0555	0.0001
Adhesiveness (N×s)	T_1	Intercept	-0.473	0.0692	0.0001	-1.650	0.1111	0.0001	-0.3272	0.0498	0.0001	-1.0976	0.0886	0.0001
		Slope	0.001	0.0002	0.0001	0.003	0.0002	0.0001	0.0004	0.0001	0.0001	0.0015	0.0001	0.0001
	T_2	Intercept	-0.406	0.0647	0.0001	-0.818	0.0829	0.0001	0.0438	0.0160	0.0085	-0.0001	0.0364	0.9977
		Slope	0.005	0.0013	0.0002	0.018	0.0023	0.0001	-0.0022	0.0003	0.0001	-0.0053	0.0009	0.0001
Springiness (m)[e]	T_1	Intercept	4.8800	0.2957	0.0001	8.7200	0.6383	0.0001	5.4800	0.7527	0.0001	7.0800	0.4625	0.0001
		Slope	-0.0092	0.0007	0.0001	-0.0144	0.0014	0.0001	-0.0073	0.0013	0.0001	-0.0065	0.0008	0.0001
	T_2	Intercept	4.0100	0.3521	0.0001	5.4300	0.3618	0.0001	-0.4916	0.2710	0.0753	2.1600	0.1275	0.0001
		Slope	-0.0582	0.0073	0.0001	-0.0893	0.0098	0.0001	0.0321	0.0052	0.0001	0.0299	0.0033	0.0001
Cohesiveness	T_1	Intercept	-0.423	0.1076	0.0003	-1.377	0.1070	0.0001	-1.207	0.1972	0.0001	-0.229	0.0302	0.0001
		Slope	0.002	0.0003	0.0001	0.004	0.0002	0.0001	0.003	0.0003	0.0001	0.001	0.0001	0.0001
	T_2	Intercept	-0.166	0.1193	0.1697	-0.487	0.0528	0.0001	1.159	0.0551	0.0001	0.315	0.0150	0.0001
		Slope	0.014	0.0025	0.0001	0.027	0.0014	0.0001	-0.014	0.0011	0.0001	-0.003	0.0004	0.0001

[a] Cheese variety (I-CLM, T-CLM, I-CL, and T-CL) according to Table S1.

[b] SD = standard deviation; [c] RT = ripening time; [d] WC = water content; [e] Values of Intercept and Slope for Springiness are $\times 10^3$.

Table S4. Simple linear regression analysis of ripening time, water content and a_w with Apparent Diffusion Coefficient (*ADC*) of the four varieties of Spanish ewe´s milk cheese.

Dependent variable	Cheese variety[a] and Independent variables		Regression equation coefficients	*p*-value[b]	R^2	SE[c]
**RT (days)**	I-CLM	Intercept	149.46	0.0001	0.44***	50.67
		Slope	-137.21	0.0001		
	T-CLM	Intercept	187.31	0.0001	0.49***	48.39
		Slope	-193.01	0.0001		
	I-CL	Intercept	139.33	0.0001	0.42***	51.76
		Slope	-131.17	0.0001		
	T-CL	Intercept	200.14	0.0001	0.54***	46.29
		Slope	-187.41	0.0001		
**WC (%)**	I-CLM	Intercept	31.43	0.0001	0.73**	3.660
		Slope	16.07	0.0010		
	T-CLM	Intercept	32.93	0.0001	0.47*	3.400
		Slope	16.02	0.0050		
	I-CL	Intercept	28.74	0.0001	0.69**	4.330
		Slope	18.96	0.0001		
	T-CL	Intercept	32.88	0.0001	0.58**	2.720
		Slope	12.25	0.0010		
a_w	I-CLM	Intercept	0.93	0.0001	0.67**	0.005
		Slope	0.02	0.0002		
	T-CLM	Intercept	0.90	0.0001	0.42*	0.020
		Slope	0.06	0.0300		
	I-CL	Intercept	0.90	0.0001	0.46*	0.020
		Slope	0.06	0.0060		
	T-CL	Intercept	0.87	0.0001	0.58**	0.020
		Slope	0.08	0.0010		

[a] Cheese variety (I-CLM, T-CLM, I-CL and T-CL) according to Table S1.

[b] ***$p < 0.0001$; **$p < 0.001$; * $p < 0.01$.

[c] SE = standard error of the estimate; R^2 = coefficient of determination.

Figure S1. Cheese sampling

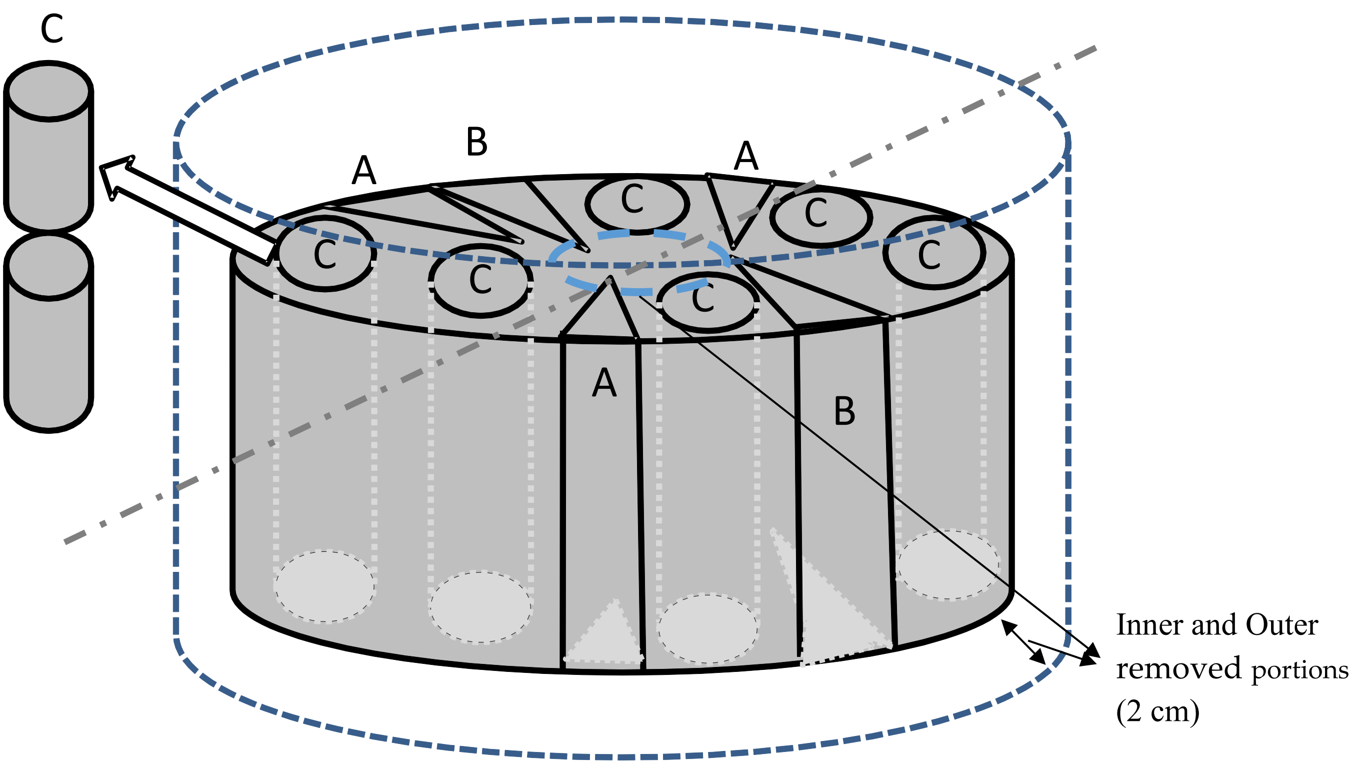

A: Samples for physicochemical analysis

B: Samples for Magnetic Resonance Imaging analysis

C: Samples for textural analysis

Figure S2. Proton Density images of the four varieties of Spanish sheep cheese at different ripening times, used to calculate the hole index.

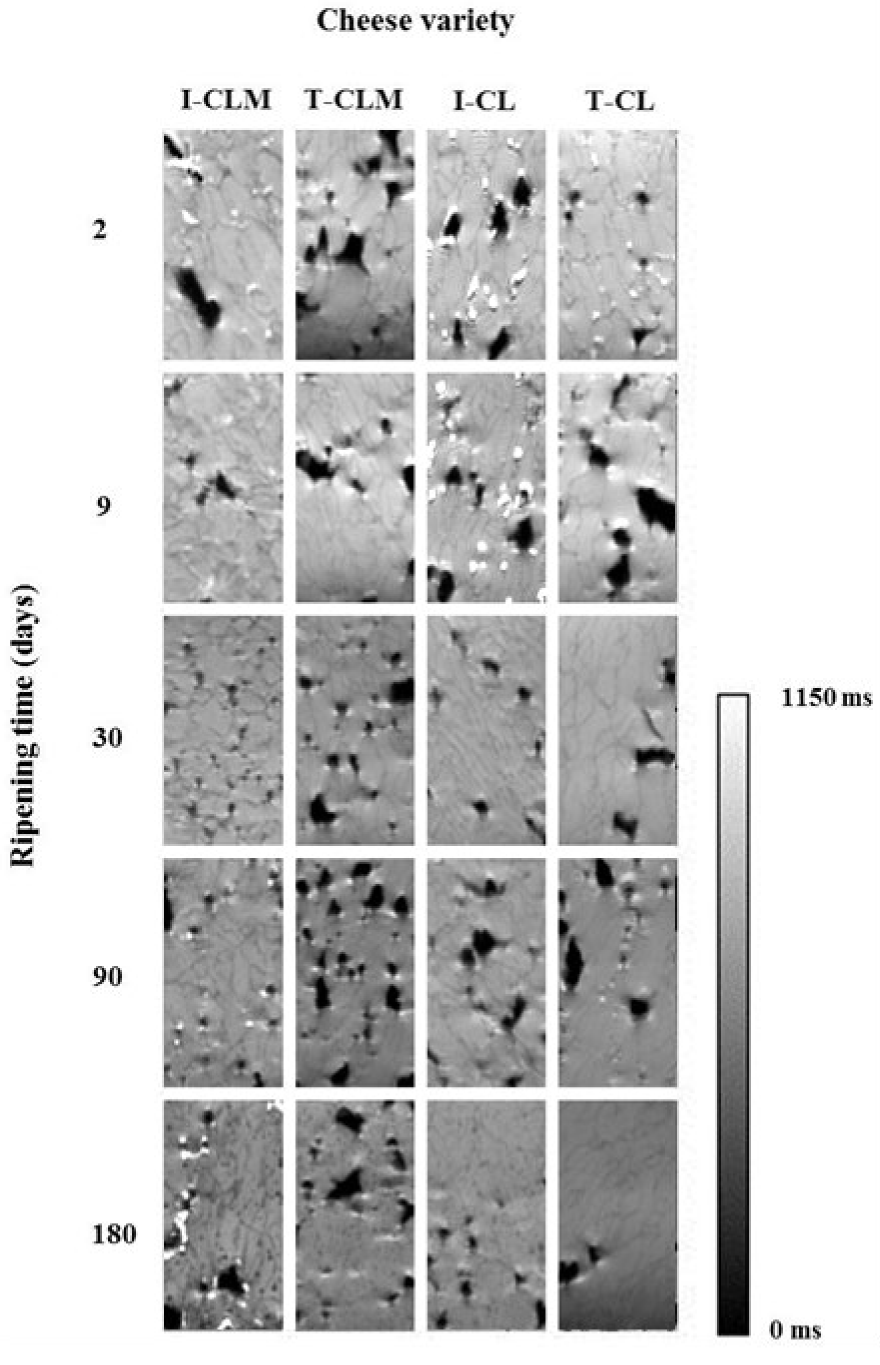

Cheese varieties (I-CLM, T-CLM, I-CL, and T-CL) according to Table S1

Figure S3. Response surface plot showing the relationship between T_1 and T_2 values (MRI parameters), and physicochemical parameters: a) ripening time, RT (days); b) water activity, a_w; c) water content, WC (%) of the four varieties of Spanish sheep cheese.

a) RT **I-CLM** **T-CLM** **I-CL** **T-CL**

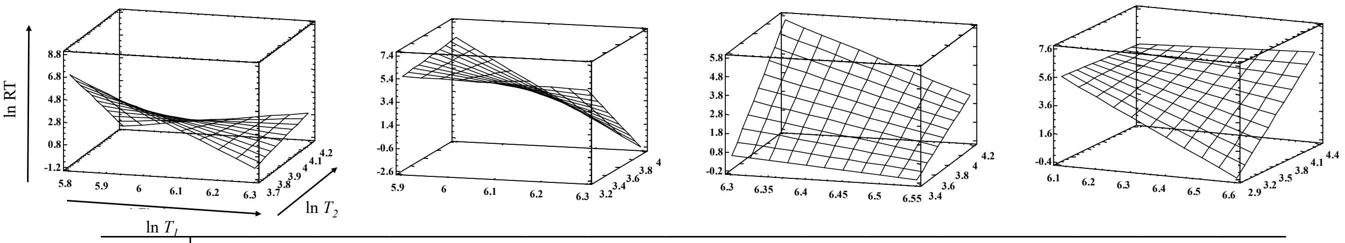

	I-CLM[a]				T-CLM				I-CL				T-CL			
**Coefficient**	lnT_1	lnT_2	lnT_1 lnT_2	Intercept	lnT_1	lnT_2	lnT_1 lnT_2	Intercept	lnT_1	lnT_2	lnT_1 lnT_2	Intercept	lnT_1	lnT_2	lnT_1 lnT_2	Intercept
**Value**	-152.7	-232.8	37.37	951.6	81.82	153.4	-25.88	-479.5	53.64	105.3	-15.65	-360.4	-37.36	-53.71	8.77	234.1
**SD[b]**	36.64	59.47	9.667	225.3	14.40	24.39	4.002	87.56	18.51	32.02	5.019	118.3	6.400	11.10	1.750	41.07
**P-value**	0.0001	0.0002	0.0003	0.0005	0.0001	0.0001	0.0001	0.0009	0.0057	0.0019	0.0031	0.0017	0.0001	0.0001	0.0001	0.0001

b) a_w

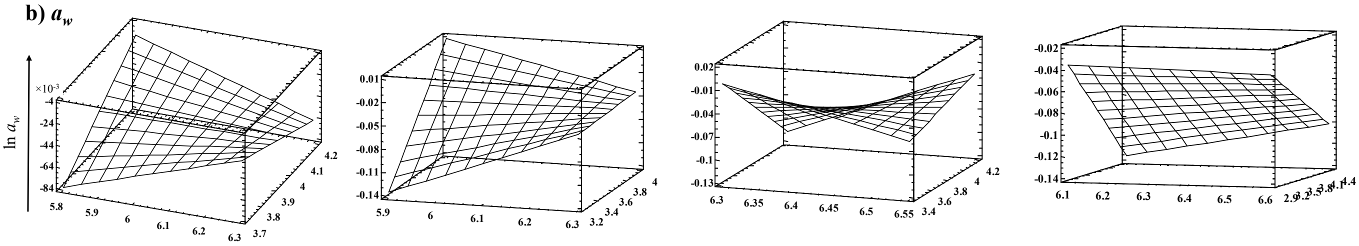

	I-CLM				T-CLM				I-CL				T-CL			
**Coefficient**	lnT_1	lnT_2	lnT_1 lnT_2	Intercept	lnT_1	lnT_2	lnT_1 lnT_2	Intercept	lnT_1	lnT_2	lnT_1 lnT_2	Intercept	lnT_1	lnT_2	lnT_1 lnT_2	Intercept
**Value**	1.64	2.54	-0.41	-10.10	1.66	2.82	-0.45	-10.49	-2.76	-4.83	0.75	17.90	-0.15	-0.38	0.05	1.10
**SD**	0.489	0.795	0.129	3.005	0.289	0.493	0.081	1.757	1.418	1.385	0.274	8.052	0.121	0.209	0.033	0.774
**P-value**	0.0025	0.0036	0.0036	0.0024	0.0001	0.0001	0.0001	0.0001	0.0101	0.0131	0.016	0.0135	0.0073	0.0066	0.0056	0.0081

c) WC

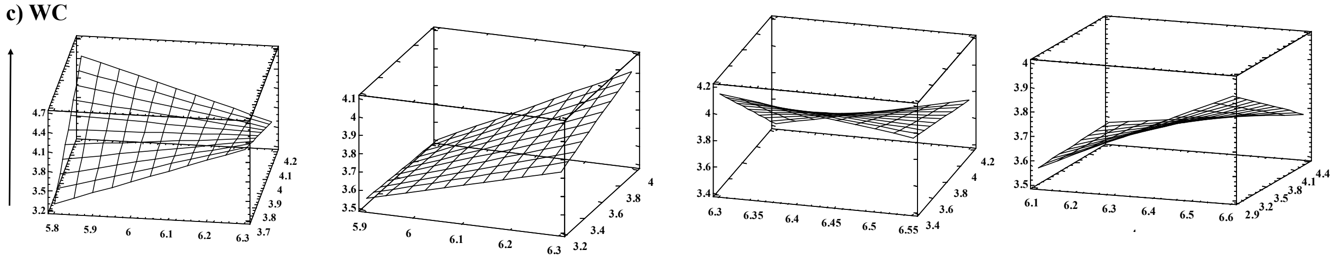

	I-CLM				T-CLM				I-CL				T-CL			
**Coefficient**	lnT_1	lnT_2	lnT_1 lnT_2	Intercept	lnT_1	lnT_2	lnT_1 lnT_2	Intercept	lnT_1	lnT_2	lnT_1 lnT_2	Intercept	lnT_1	lnT_2	lnT_1 lnT_2	Intercept
**Value**	31.38	48.39	-7.91	-188.1	-4.410	-8.920	1.470	30.40	-8.345	-15.20	2.248	60.29	1.726	2.142	-0.349	-6.995
**SD**	7.195	11.697	1.903	44.21	3.182	5.605	0.919	19.98	2.469	4.240	0.664	15.77	0.386	0.669	0.105	2.478
**P-value**	0.0002	0.0003	0.0003	0.0009	0.0003	0.0009	0.0013	0.0005	0.0023	0.0014	0.0023	0.0095	0.0001	0.0036	0.0027	0.0099

[a]Cheese variety (I-CLM, T-CLM, I-CL and T-CL) according to Table S1
[b]SD = standard deviation

Figure S4. Response surface plot showing the relationship between T_1 and T_2 values (MRI parameters) and texture parameters: a) Hardness (N), b) Adhesiveness (N×s), c) Springiness (m), and d) Cohesiveness, of the four varieties of Spanish sheep cheese.

I-CLM **T-CLM** **I-CL** **T-CL**

a) Hardness (N)

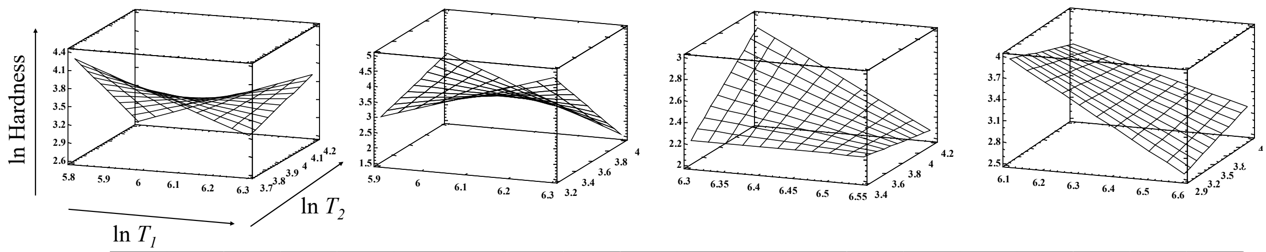

	I-CLM[a]				T-CLM				I-CL				T-CL			
**Coefficient**	Intercept	lnT_1	lnT_2	lnT_1 lnT_2	Intercept	lnT_1	lnT_2	lnT_1 lnT_2	Intercept	lnT_1	lnT_2	lnT_1 lnT_2	Intercept	lnT_1	lnT_2	lnT_1 lnT_2
**Value**	434.4	-69.75	-111.8	18.08	-308.5	52.20	86.58	-14.50	-178.6	28.31	52.23	-8.152	65.31	-9.459	-10.14	1.508
**SD[b]**	63.46	10.320	16.752	2.7229	32.25	5.303	8.986	1.474	45.78	7.162	12.103	1.8960	9.671	1.5070	2.614	0.4120
**P-value**	0.0001	0.0001	0.0001	0.0001	0.0001	0.0001	0.0001	0.0001	0.0003	0.0003	0.0001	0.0001	0.0001	0.0001	0.0003	0.0006

b) Adhesiveness (N×s)

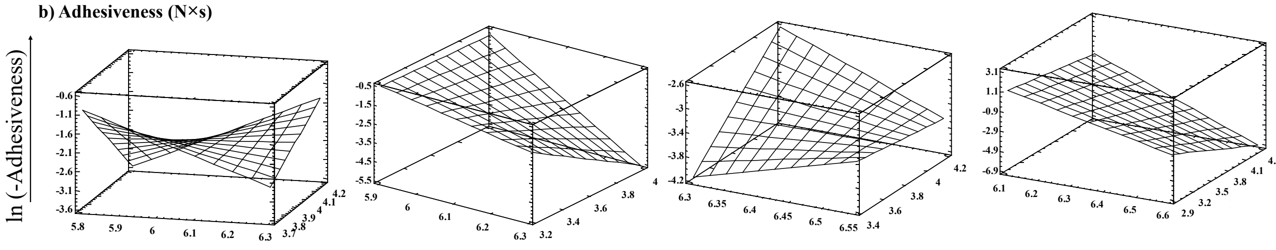

	I-CLM				T-CLM				I-CL				T-CL			
**Coefficient**	Intercept	lnT_1	lnT_2	lnT_1 lnT_2	Intercept	lnT_1	lnT_2	lnT_1 lnT_2	Intercept	lnT_1	lnT_2	lnT_1 lnT_2	Intercept	lnT_1	lnT_2	lnT_1 lnT_2
**Value**	877.8	-143.6	-227.0	37.04	-226.6	37.66	79.74	-13.31	-297.7	45.22	78.10	-11.99	135.3	-21.25	-26.54	4.095
**SD**	178.02	28.95	46.99	7.638	104.5	17.174	29.100	4.774	74.46	11.648	19.683	3.083	26.14	4.074	7.067	1.1138
**P-value**	0.0001	0.0001	0.0001	0.0001	0.0352	0.0334	0.0087	0.0077	0.0002	0.0003	0.0003	0.0003	0.0001	0.0001	0.0005	0.0006

c) Springiness (m)

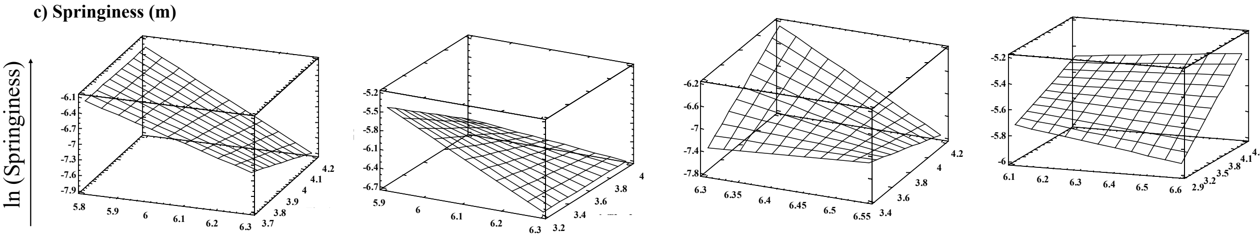

	I-CLM				T-CLM				I-CL				T-CL			
**Coefficient**	Intercept	lnT_1	lnT_2	lnT_1 lnT_2	Intercept	lnT_1	lnT_2	lnT_1 lnT_2	Intercept	lnT_1	lnT_2	lnT_1 lnT_2	Intercept	lnT_1	lnT_2	lnT_1 lnT_2
**Value**	-87.95	13.44	26.27	-4.357	83.88	-14.44	-21.71	3.469	-198.8	29.63	54.69	-8.460	5.638	-1.956	-2.784	0.486
**SD**	7.644	4.002	7.995	0.8728	19.313	2.4636	5.9563	0.7973	14.67	3.251	9.426	3.514	0.5585	0.4852	0.5295	0.0371
**P-value**	0.0343	0.0373	0.0088	0.0292	0.0377	0.0299	0.023	0.0392	0.0228	0.0298	0.0183	0.0197	0.0184	0.0248	0.0272	0.0274

d) Cohesiveness

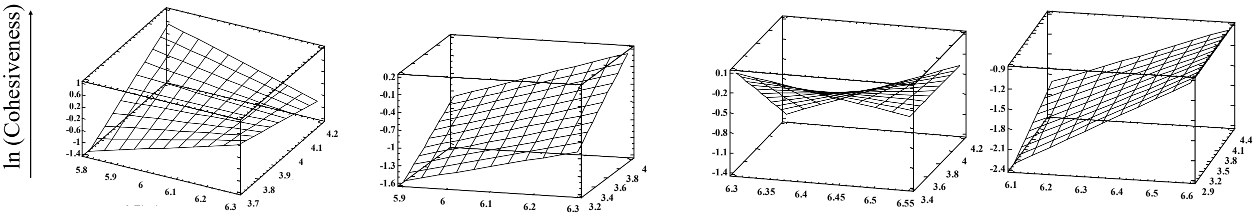

	I-CLM				T-CLM				I-CL				T-CL			
**Coefficient**	Intercept	lnT_1	lnT_2	lnT_1 lnT_2	Intercept	lnT_1	lnT_2	lnT_1 lnT_2	Intercept	lnT_1	lnT_2	lnT_1 lnT_2	Intercept	lnT_1	lnT_2	lnT_1 lnT_2
**Value**	-344.1	56.21	86.73	-14.18	-16.32	1.851	0.929	0.041	161.6	-24.72	-44.38	6.769	-39.20	5.693	4.649	-0.670
**SD**	59.68	12.96	21.03	3.4188	1.088	0.3385	0.0318	0.0024	28.47	9.146	10.456	1.4211	7.0488	1.0008	1.0135	0.0317
**P-value**	0.0001	0.0001	0.0002	0.0001	0.0397	0.0282	0.0321	0.0223	0.0082	0.0096	0.0062	0.0075	0.0001	0.0001	0.0255	0.0403

[a]Spanish sheep cheese variety (I-CLM, T-CLM, I-CL and T-CL) according to Table S1
[b]SD = standard deviation
